# Prevalence of risk factors for breast cancer in German airline cabin crew: a cross-sectional study

**DOI:** 10.1186/1745-6673-9-27

**Published:** 2014-07-17

**Authors:** Mareen Winter, Maria Blettner, Hajo Zeeb

**Affiliations:** 1 Institute for Medical Statistics, Epidemiology and Informatics, University Medical Center, Johannes Gutenberg University Mainz, Obere Zahlbacher Straße 69, 55131 Mainz, Germany; 2 Leibniz Institute for Prevention Research and Epidemiology-BIPS, Dep. of Prevention and Evaluation, Achterstr. 30, 28359 Bremen, Germany; 3 Health Sciences Bremen, University of Bremen, Germany, Bibliothekstr 1, 28359 Bremen, Germany

**Keywords:** Breast cancer, Cabin crew, Flight attendants, Germany

## Abstract

**Background:**

Many epidemiological studies point to an increased risk of breast cancer among female airline cabin crew. Possible causes include occupational factors (e.g. cosmic radiation exposure, chronodisruption), as well as lifestyle and reproductive factors.

**Aims:**

To investigate the frequency of various risk factors in German flight attendants which are recognised to be associated with breast cancer.

**Methods:**

2708 current and former female cabin crew were randomly selected by a flight attendants’ union and mailed a questionnaire; 1311 responded (48% response). Descriptive statistics were used to compare the distribution of breast cancer risk factors with general German population data.

**Results:**

On average, cabin crew were 3.0 cm (95% CI 2.7-3.3) taller than the comparison group, while their body mass index was 2.5 kg/m^2^ (95% CI 2.4-2.6) lower. We found less use of hormone replacement therapy, but longer average use of oral contraceptives. Nulliparity among respondents aged 45+ was 57% (95% CI 54%-60%) compared to 16%. Average age at first birth was 32.1 years (95% CI 31.7-32.4) vs. 25.5 years. The birth rate was 0.62 (95% CI 0.58-0.67), less than half the population average of 1.34. Alcohol consumption was considerably higher, whereas cabin crew tended to smoke less and performed more physical exercise.

**Conclusion:**

We found important differences in terms of anthropometric, gynaecological, reproductive and lifestyle factors. Some of these differences (e.g. higher nulliparity, alcohol consumption, taller size) could contribute to a higher breast cancer risk, whereas others could lead to a reduction (e.g. increased physical exercise, lower BMI, less HRT use).

## Introduction

Breast cancer is currently the most frequently diagnosed form of cancer in women in Germany, accounting for 31% of incident cancer cases in 2010 [1]. The question whether female cabin crew have a higher risk of breast cancer than other women has been the subject of several studies in recent years. A significant increase of breast cancer incidence among female flight attendants has been found in several countries, as shown by studies from Finland, Denmark, Iceland and the USA [2-6]. A smaller, non-significant increase was found by two studies from Norway and Sweden [7,8]. A meta-analysis of incidence studies published until 2005 yielded a combined relative risk of 1.41 (95% CI 1.22-1.62) [9], consistent with an earlier meta-analysis by Buja et al. [10]. Recently, a 50% risk increase was reported from Nordic female cabin crew [11]. Current data on mortality from breast cancer among cabin crew were reported from a pooled analyses of US and European cohort studies, and in contrast to the incidence studies, no significant increase was found [12].

The causes of increased breast cancer incidence in this group have not been clearly established: few studies on this topic exist, showing inconsistent results. In particular, it is unclear to what extent occupational factors (e.g. exposure to cosmic radiation during flights, jet lag) play a role versus other factors, e.g. lifestyle or reproductive factors. Tokumaru et al. claim that 29% of breast cancer in flight attendants may be attributable to their occupation [9]. An Icelandic study also indicates that occupational factors may be an important cause of breast cancer among cabin attendants [13]. No association between occupational factors and breast cancer, however, has been found by studies from Sweden and Finland [7,14]. In these studies the increased incidence was explained by risk factors that can also be found in the general population.

We conducted a cross-sectional study of current and retired cabin crew in Germany. The aim was to investigate the distribution of several known and suspected breast cancer risk factors among this group and perform comparisons to reported prevalences in the general female German population of similar age.

## Methods

The source population consisted of current and retired female cabin crew who were resident in Germany, had served at least one year of active duty and were member of the German Cabin Crew Union “Unabhängige Flugbegleiter Organisation” (UFO) between 2000 und 2004. A total of 7616 eligible women were identified from the membership database. A questionnaire was sent to 3000 randomly selected members. 292 letters were returned undeliverable, which yielded 2708 successfully mailed questionnaires. A reminder postcard was sent after one month to all 2708 adresses. All responses were anonymous, and thus only fully anonymous data was available for the analysis.

Information was collected on: a.) demographic factors including age, marital status, education; b.) anthropometric factors including height and weight; c.) occupational factors including service history, disturbances related to disruption of circadian rhythm, exposure to pesticides; d.) lifestyle factors including alcohol consumption, smoking and physical exercise; e.) gynaecological factors including menstruation, use of hormone replacement therapy and contraceptives; f.) reproductive factors including pregnancies, child birth, miscarriages and abortions, breast feeding; g.) family history of breast cancer, participation in screening programmes; h.) general health status. This paper focuses on a subset of the information collected: specifically anthropometric, lifestyle, gynaecological and reproductive factors, for which we found clear differences between cabin crew and the general population.

All questionnaires were captured electronically and responses were coded into a database. The participant’s age was calculated as 2009 – year of birth (the study period was Nov 2008 through March 2009). We calculated the body mass index (BMI) based on the formula weight (in kg) divided by height (in metres) squared. Alcohol consumption was measured in grams per week, based on reported consumption in multiples of 0.33 litres of beer, 0.25 litres of wine, 2 cl of spirits, and average alcohol content.

Data from our study was then compared to general population data, which was taken from two main sources: Firstly, data on birth statistics, abortions and miscarriages, anthropometrics, marital status and education was supplied directly by the German Federal Statistical Office; secondly, the Robert Koch-Institute (RKI) provided data on lifestyle factors (alcohol consumption, smoking, physical exercise), hormonal and gynaecological factors, general health status [15].

Since the age composition of our study group was very different from the general population, it was necessary to control for age. To this end, we stratified the sample into 5-year age groups from 25–29 up to 65–69 years. For each risk factor, we calculated relative frequencies (for categorical variables) or averages (for metric variables) and 95% confidence intervals, by age group and for the total study population. This was compared to general population data, both by comparing each individual 5-year age group and by calculating age-adjusted averages, where we weighted each age group in the general population with the frequency of our study population.

Ethical approval for the study was obtained from the ethical review board of the medical chamber Rhineland-Palatinate, Mainz, Germany.

## Results

A total of 1311 flight attendants returned the questionnaire (n = 1310 valid responses), yielding a response rate of 48%. Participants were between 25 and 69 years old (except for two respondents aged 70 and 71, respectively) with a mean age of 41.6 years.

Women in our study were on average 169.3 cm tall (95% CI 169.0-169.6), that is 3.0 cm taller compared to the general population. This applied consistently across age strata. The average BMI was 21.8 kg/m^2^ (95% CI 21.7-21.9), significantly lower than the corresponding population average of 24.3 kg/m^2^.

Use of oral contraceptives (OC) was widespread among cabin crew; 96% of respondents indicated that they had ever used OC, for an average duration of 11.1 years (95% CI 10.7-11.5), which is 2.0 years longer than the population average. We also investigated the use of hormone replacement therapy (HRT). 23% (95% CI 15%-31%) of 50–54 year old flight attendants have “ever used” such treatment. Unfortunately, suitable data for comparison were lacking. A study by the Robert Koch-Institute, however, indicated that 33% of 50–54 year old and 18% of 45–49 year old women “currently” use HRT. This suggests that the use of HRT among cabin crew could be lower than for the general female public in the respective age group.

68% (95% CI: 65%–70%) of flight attendants had performed regular physical activity in the last three months, only slightly more than the population average of 63.7; however, at 3.9 hours/week (95% CI 3.7-4.0), they exercised 48% longer. Alcohol consumption was significantly increased: 42% vs. 15% of respondents consume more than 10 g of alcohol daily. Average consumption was 73.9 g per week (95% CI 69.3-78.5), i.e. 10.6 g daily. This compares to a typical daily consumption rate of 6.0 g in industrialised countries [16]. Reported consumption was particularly high among the older cohorts, e.g. 107.9 g (95% CI 92.5-123.4) for 50–59 year olds. We also asked cabin crew about their consumption 10 years ago, but found no significant differences vs. current consumption levels. Smoking appeared less common among cabin crew: Only 13% vs. 28% smoked daily, with the average consumed quantity among smokers also reduced at 7.9 (95% CI 7.0-8.7) vs. 12.7 cigarettes per day. There were no significant differences, however, for the age when people first started smoking, or for the percentage of people who have ever smoked daily at some point in the past. Table 1 summarizes main survey findings.

**Table 1:** Comparison of breast cancer risk factors in cabin crew vs. the general population

**Risk factor**	**Study population (95% confidence interval)**	General population*
Body height (cm)	169.3 (169.0-169.6)	166.3
Body mass index (kg/m^2^)	21.8 (21.7-21.9)	24.3
Duration of OC use (years)	11.1 (10.7-11.5)	9.1
Physical activity (h/week)	3.9 (3.7-4.0)	2.6
*Alcohol consumption*		
% consuming over 10 g/day	41.9	14.5
Avg. daily consumption (g)	10.6 (9.9-11.2)	6.0
*Smoking*		
% of daily smokers	12.6	28.1
Avg. cigarettes per day	7.9 (7.0-8.7)	12.7
*Reproductive factors*		
Nulliparity^1^ (%)	57.1 (54.4-59.8)	16.1
Age at first birth (years)	32.1 (31.7-32.4)	25.5
Birth rate (no. of children)	0.62 (0.58-0.67)	1.34

*age adjusted.

^1^) only considering women aged 45 years and older.

Cabin crew reported differences in reproductive history compared to the general population. 57% (95% CI 54%-60%) of cabin crew aged 45 years and older were nulliparous, compared to only 16%. Those with offspring tended to have their first birth with 32.1 years (95% CI 31.7-32.4), 6.6 years older than the population average. Overall, the birth rate among the study group was only 0.62 (95% CI 0.58-0.67), 54% lower than the corresponding population average of 1.34.

## Discussion

Earlier studies showed a significant increase in breast cancer incidence among female flight attendants in several countries [2-6,9,10]. Some studies attribute this increase, at least partially, to occupational risk factors, e.g. exposure to cosmic radiation during flights, pesticides, or jet lag [9,13]. Other studies, however, conclude that this increase is likely to be a confounding effect and related to general, well-established risk factors, which also affect the general population [7,14]. In our cross-sectional study, we examined the distribution of general risk factors, which apply to both the study group and the general population, and drew comparisons between the two. We found significant differences, some of which could help to explain an increase in breast cancer incidence among cabin crew. Findings including the higher frequency of nulliparity, the lower birth rate, the higher age at first birth, taller size, higher alcohol consumption and longer use of oral contraceptives can be interpreted in this line. On the other hand though, we also found protective factors that would suggest a reduction of breast cancer risk for flight attendants: e.g. lower BMI, less use of HRT, higher levels of physical activity, less smoking.

In terms of anthropometric differences, we found that flight attendants were on average taller than the general female population, while their BMI was lower. This difference could be explained by entry selection criteria for the occupational group. Height is a well-established risk factor for breast cancer: both pre- and postmenopausal risk increase with height. A pooled analysis found a relative risk (RR) of 1.02 (95% CI: 0.96-1.10) for pre- and 1.07 (95% CI 1.03-1.12) for post-menopausal breast cancer per 5 cm difference in height [15]. A higher BMI has been recognised as a protective factor for pre-menopausal breast cancer, but a risk factor for post-menopausal tumour occurrence [16-18]; therefore we would estimate an increased risk of 4% for pre-menopausal cancer and a reduced risk of 10% for post-menopausal cancer for cabin crew.

In addition, we found higher than average usage levels of oral contraceptives by flight attendants. This finding is consistent with the result that cabin crew are more likely to remain nulliparous or have their first birth at a higher age than the average population. The impact of oral contraceptives on breast cancer risk is not clearly established; some studies suggest that there may be a small increase of risk [19,20]. Hormone replacement therapy, on the other hand, is clearly associated with an increase in breast cancer risk [21,22]. Since flight attendants appear to receive HRT less often, this could contribute to a reduction in risk. Due to the limited comparability of our results, we are however unable to quantify an effect on risk.

We also discovered significant differences between female cabin crew and the general population for three lifestyle factors: alcohol consumption, smoking and physical exercise. Increased alcohol consumption, as we found among our study group, has been recognised as an important independent driver of breast cancer risk (RR 1.071, 95% CI 1.055-1.087, per 10 g of daily consumption) [16]. Contrary to this, reduced levels of smoking among cabin crew might act as a protective factor – this is, however, not clearly supported by current research [16,23-25]. Cabin crew might also benefit from increased physical activity, which has been reported as a protective factor [22,26].

Nulliparity and age of the mother at first birth are strong, well-recognised breast cancer risk factors. According to a meta-analysis, nulliparous women have a 30% higher risk compared to those with offspring [27]. Similarly, the mother’s age at first birth (32.1 years) was much higher than the population average (25.5 years). Women who have their first birth between age 32–34 have been estimated to incur a 40%-50% higher breast cancer risk compared to women who first deliver before the age of 28 [28]. Given the extremely high level of nulliparity and late age of first birth among our study group, we estimate this to be a strong contributor to increased breast cancer incidence, possibly explaining a risk increase of 30% or higher. Studies in Finland and Iceland yielded less marked differences. In a nested case–control study, Kojo et al. found a negligible effect of parity on breast cancer (OR = 1.10, 95% CI: 0.23-4.85) [14]. This result is not in contradiction to our findings due to the width of the confidence interval. While Rafnsson et al. agree with the view that the strongest factors that have been shown empirically to affect risk of breast cancer among occupational or social groups are parity and age of first child birth, they conclude that in their study the increased risk of breast cancer is unlikely to be explained solely by confounding due to parity [5].

Chronodisruption, although not specifically assessed in this analysis, has been discussed as a potential contributor to breast cancer risk in populations such as aircrew exposed to shift work and/or repeated time zone changes [29]. Shift work involving disruption of the circadian rhythm was classified as a possible carcinogen (IARC group 2A) in 2007 [30]. Erren and Morfeld [31] discuss the influence of potential chronotype errors in epidemiological studies and provide detailed information on appropriate inclusion of chronotype data in epidemiological studies. For our current survey, no such data were available.

Although we found rather clear differences between female cabin crew and the general population, we are aware that we compared data from different sources (e.g. official census data with self-reported data) and the methods of measurement may have been different. The above results also need to be viewed in light of the limitations of our study. Firstly, our study design as a cross-sectional study does not give insight into causal relationships between breast cancer incidence and risk factors. All conclusions therefore need to be viewed with caution, and further research is needed to validate these findings. Secondly, due to our choice of study population (mostly women of working age) there is a bias towards younger women: only n = 35 are 60 years or older, the maximum age is 72. Therefore, our study includes only a small sample of older retired cabin crew, for whom breast cancer risk and prevalence is likely to be particularly high. Thirdly, we need to consider non-response bias, which is likely to exist in our study because women, who are affected by breast cancer (either personally or in their family) might have a higher probability to respond. This could on the one hand lead to individuals with higher than average breast cancer risk to be over-represented in the sample. On the other hand, the study does of course not include cases who suffered from breast cancer and subsequently died. Finally, it should be noted that survey participants were all union members. This may introduce some selection bias in principle, but we believe that union membership is not likely to be strongly associated with health aspects, and union membership is common in this occupational group.

At the same time, there are clear strengths to our study. Firstly, the study is the first of its kind in Germany, making use of the database of a large union of flight attendants. Secondly, at 48% the response rate was rather high and exceeded our expectations during study design. Nevertheless, more than half of all contacted flight attendants chose not to reply, inducing a non-negligible potential for selection bias.

In summary, we found several factors among the study group that might help explain an increased incidence of breast cancer among the occupational group: higher nulliparity, later first birth, higher levels of alcohol consumption, a taller physique, (possibly) oral contraceptive use, and (for pre-menopausal breast cancer only) a lower BMI. In contrast, we also found factors which would lead to a reduction of risk: Lower use of hormone replacement therapy, higher levels of physical exercise, (possibly) lower levels of smoking and (for post-menopausal breast cancer only) a lower BMI. Within the scope of this study it is not possible to assess the combined effect of these factors, and to clarify to what degree risk increases are outweighed by risk reductions. We did, however, see marked differences in some reproductive and lifestyle factors, and the role of these factors in breast cancer has been recognized in many studies.

## Competing interests

The authors declare that they have no competing interests.

## Authors’contributions

MW, MB and HZ jointly designed the study. MW wrote the study protocol and conducted the data acquisition together with HZ. MW did the statistical analysis and wrote the first draft of the paper. MB supervised the data analysis. All authors contributed the interpretation of data, revised the manuscript, read and approved the final version.
